# Human Insulin Therapy Is Associated With an Increased Risk of Lung Cancer: A Population-Based Retrospective Cohort Study

**DOI:** 10.3389/fendo.2019.00443

**Published:** 2019-07-11

**Authors:** Chin-Hsiao Tseng

**Affiliations:** ^1^Department of Internal Medicine, National Taiwan University College of Medicine, Taipei, Taiwan; ^2^Division of Endocrinology and Metabolism, Department of Internal Medicine, National Taiwan University Hospital, Taipei, Taiwan; ^3^Division of Environmental Health and Occupational Medicine, National Health Research Institutes, Zhunan, Taiwan

**Keywords:** retrospective cohort study, insulin, lung cancer, type 2 diabetes mellitus, Taiwan

## Abstract

**Background:** Whether human insulin may affect lung cancer risk requires investigation.

**Methods:** All patients with a diagnosis of diabetes mellitus from 1996 to 2009 were enrolled from Taiwan's National Health Insurance. An entry date was set on January 1, 2004, and 1,007,617 patients with type 2 diabetes mellitus diagnosed before 2004 were followed up for new-onset lung cancer until December 31, 2009. Incidence rates of lung cancer for never-users, ever-users, and tertiles of three dose-response exposure parameters (i.e., time since starting insulin, cumulative dose, and cumulative duration) were calculated. Adjusted hazard ratios were estimated by Cox proportional hazards models. The joint effect of insulin and chronic obstructive pulmonary disease was also evaluated.

**Results:** There were 156,720 ever-users and 850,897 never-users. The respective case numbers of incident lung cancer were 3,007 (1.92%) and 13,677 (1.61%), and the respective incidence rates were 424.45 and 313.60 per 100,000 person-years. The adjusted hazard ratio comparing ever-users vs. never-users was 1.545 (95% confidence interval: 1.478–1.614). The hazard ratios for the different subgroups of the three dose–response parameters all suggested a significantly higher risk of lung cancer associated with insulin use (*P* trend < 0.0001). Compared to patients without insulin use and without chronic obstructive pulmonary disease, insulin users who also had chronic obstructive pulmonary disease had the highest risk of lung cancer (adjusted hazard ratio: 1.891, 95% confidence interval: 1.767–2.024).

**Conclusions:** This study suggests a significant association between human insulin use and lung cancer risk in patients with type 2 diabetes mellitus.

## Introduction

The insulin-like growth factor (IGF) system, involving two hormones of IGF-1 and IGF-2, two receptors to these two hormones (IGF-1R and IGF-2R), and several binding proteins (IGFBP1-7), plays an important role in the development of lung cancer (1, 2). Normally, IGF-1 and IGF-2 are produced by the liver, and they share sequence homology with insulin, which is produced by the pancreas (1). There are two types of insulin receptors (IRs), i.e., IR-A (fetal isoform) and IR-B (mature isoform) (2). While IR-B is mainly involved in glucose homeostasis, IR-A activation by insulin or IGF-2 may lead to cell proliferation and tumorigenesis (3). IGF-1 may also bind to IR-A, but to a lesser extent of approximately one-tenth affinity of insulin or IGF-2 (2). Lung cancer cells express receptors for insulin (mainly IR-A) and IGF-1 (2) and insulin can activate IR-A and IGF-1R leading to the proliferation of cancer cells (4). Therefore, hyperinsulinemia resulting from either insulin resistance or prolonged insulin administration for the treatment of diabetes may potentially lead to the development of lung cancer.

In a UK study, insulin therapy with or without oral anti-diabetic drugs was not associated with lung cancer (5). However, another study recruiting postmenopausal women in the Women's Health Initiative study in the USA suggested that diabetes increased the risk of lung cancer (hazard ratio: 1.27, 95% confidence interval: 1.02–1.59), which was more remarkable among patients treated with insulin (hazard ratio: 1.71, 95% confidence interval: 1.15–2.53) (6). Two meta-analyses also gave different conclusions. While Wu et al. (7) concluded that insulin was associated with an increased risk of lung cancer in patients with diabetes mellitus (odds ratio: 1.23, 95% confidence interval: 1.10–1.35). Nie et al. (8) did not find an increased risk of lung cancer associated with insulin use (odds ratio: 1.13, 95% confidence interval: 0.79–1.62).

Because insulin is widely used for the treatment of hyperglycemia in patients with either type 1 or type 2 diabetes mellitus, it is urgently needed to clarify its potential role in lung cancer. Therefore, the present study aimed at investigating whether human insulin use would affect lung cancer risk in Taiwanese patients with type 2 diabetes mellitus.

## Materials and Methods

This is a population-based retrospective cohort study that used the reimbursement database of the Taiwan's National Health Insurance (NHI). The study was approved by an ethics review board of the National Health Research Institutes with registered approval number 99274. The database has been de-identified for the protection of privacy and informed consent was not required according to local regulations.

The NHI is a unique and universal healthcare system implemented in Taiwan since March 1995. It is compulsory and covers more than 99.6% of Taiwan's population. The Bureau of the NHI has contracts with all in-hospitals and approximately 93% of all medical settings nationwide. The database keeps records of all disease diagnoses, drug prescriptions, and clinical procedures of all insurants for each outpatient visit, use of emergency service, and hospital admission.

During the study period, diabetes mellitus was coded 250.1–250.9 and lung cancer was coded 162, based on the *International Classification of Diseases, Ninth Revision, Clinical Modification* (ICD-9-CM).

**Figure 1:**
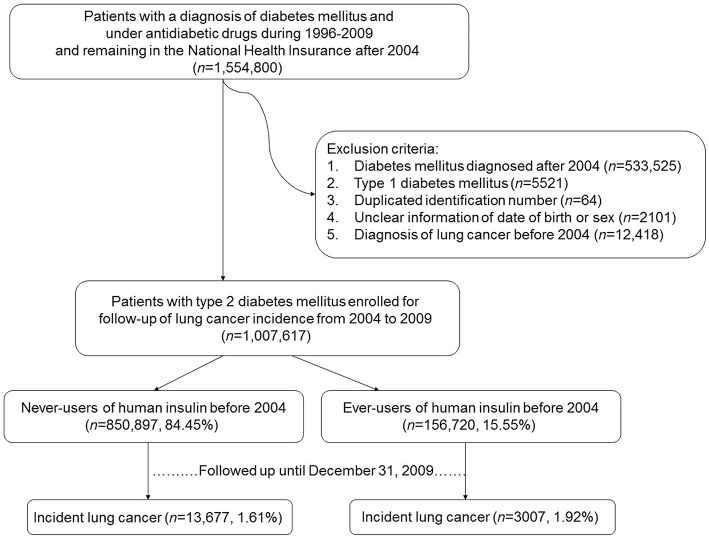
Flowchart showing the procedures used in selecting the studied patients from Taiwan's National Health Insurance database.

Figure 1 shows the procedures used in selecting patients into the study. At first, an entry date of January 1, 2004, was selected and the database of all patients who had a diagnosis of diabetes mellitus and under treatment with anti-diabetic drugs during the period of 1996–2009 and remained in the insurance program after the entry date were retrieved (*n* = 1,554,800). The entry date was so selected because of the following reasons. First, insulin analogs, which may have a different profile of cancer risk from human insulin (9, 10), were not marketed in Taiwan until after February 2004 (the first marketed was insulin glargine). Therefore, all patients enrolled into the study might not have taken any form of insulin analogs before the entry date. Second, because the information of each patient could be tracked from the time of his/her diabetes diagnosis to December 31, 2009, this entry date allowed a maximal follow-up duration of 6 years, such that enough case number of incident lung cancer and enough follow-up duration could be obtained. Third, because insulin analogs might have replaced human insulin originally used by the patients, an entry date set after the introduction of insulin analogs might have made the forms of insulin used by the patients at the time of their enrollment more complicated.

Several criteria were then applied to exclude ineligible patients, such as those who had a diabetes diagnosis after the entry date of January 1, 2004 (*n* = 533,525), patients with type 1 diabetes mellitus (*n* = 5,521; patients with type 1 diabetes mellitus in Taiwan were issued a so-called “Severe Morbidity Card” after certified diagnosis and they were waived for much of the co-payments), duplicated identification number (*n* = 64), unclear information on the date of birth or sex (*n* = 2,101), and patients having a diagnosis of lung cancer before entry date (*n* = 12,418). Finally, 1,007,617 patients were enrolled. These patients had a diagnosis of type 2 diabetes mellitus and under treatment with oral anti-diabetic drugs or human insulin and had no prevalent lung cancer at the time of enrollment.

Patients who had been prescribed insulin (only human insulin was available) before the entry date were defined as ever-users (*n* = 156,720, 15.55%). Never-users of insulin (*n* = 850,897, 84.45%) were defined as patients who had not received any insulin treatment before the entry date. A dose-response relationship was evaluated by using the tertile cutoffs of three variables: time since starting insulin in months, duration of insulin therapy in months, and cumulative dose of insulin in units.

All comorbidities and covariates were determined as a status/diagnosis before the entry date. The ICD-9-CM codes for the comorbidities were as follows: nephropathy 580–589; urinary tract disease 590–599; hypertension 401–405; COPD 490–496; stroke 430–438; ischemic heart disease 410–414; peripheral arterial disease 250.7, 785.4, 443.81, and 440–448; eye disease 250.5, 362.0, 369, 366.41, and 365.44; dyslipidemia 272.0–272.4; congestive heart failure 398.91, 402.11, 402.91, 404.11, 404.13, 404.91, 404.93, and 428; and cancer other than lung cancer 140–208 (excluding 162). Medications included pioglitazone, rosiglitazone, sulfonylurea, meglitinide, metformin, acarbose, statin, fibrate, angiotensin-converting enzyme inhibitor and/or angiotensin receptor blocker, calcium channel blocker, aspirin, and non-steroidal anti-inflammatory drugs. Because the frequency of outpatient visits might have affected the detection rate of lung cancer, the average times of outpatient visits per year were calculated and considered as a potential confounder. Baseline characteristics between ever-users and never-users of insulin were compared by Student's *t*-test for the times of outpatient visits/year and by chi-square test for all other categorical variables.

The incidence density of lung cancer was calculated for never-users, ever-users, and subgroups of the dose-response parameters of exposure to human insulin. The numerator of the incidence was the number of patients with incident lung cancer during a 6-year follow-up from January 1, 2004, to December 31, 2009. The denominator of the incidence was the person-years of follow-up. For ever-users, the follow-up duration was censored at lung cancer diagnosis, at the date of the last reimbursement record, or at the date of the initiation of insulin analogs, whichever occurred first. For never-users, the follow-up was censored at the date of lung cancer diagnosis, the last reimbursement record, or any type of insulin (either human insulin or insulin analogs) initiation, whichever occurred first. This ensured no exposure to insulin analogs in the group of ever-users of human insulin throughout the follow-up period, and no exposure to insulin of any type in the referent group of never-users during follow-up.

Cox proportional hazards regression was used to estimate the hazard ratios after adjustment for all variables compared previously as baseline characteristics. *P* trend was also calculated for the dose-response parameters.

Because COPD is a risk factor of lung cancer (11), to further explore the joint effect between insulin and COPD on lung cancer risk, adjusted hazard ratios were estimated for the following four subgroups: (1) insulin (–)/COPD (–) as referent group, (2) insulin (+)/COPD (–), (3) insulin (–)/COPD (+), and (4) insulin (+)/COPD (+).

To further exclude the potential residual confounding from COPD, the incidence rates of lung cancer in never-users and ever-users of insulin and the adjusted hazard ratios for ever-users vs. never-users of insulin were estimated in subgroups of patients with and without COPD, respectively. *P* interaction between insulin and COPD was also estimated.

Analyses were conducted using SAS statistical software, version 9.1 (SAS Institute, Cary, NC). *P* < 0.05 was considered statistically significant.

## Results

Table 1 shows the baseline characteristics in ever-users (*n* = 156,720) and never-users (*n* = 850,897) of human insulin. All variables differed significantly between the two groups. Ever-users are characterized by older age distribution, less male sex, higher proportion with a diabetes duration ≥5 years, higher proportions of all comorbidities and other cancer, higher proportions of using other medications, and more frequent outpatient visits/year.

**Table 1:** Baseline characteristics in never-users and ever-users of human insulin.

	**Human insulin**				
**Variables**	**Never-users**		**Ever-users**		***P***
	***n***	**%**	***n***	**%**	
*n =* 1,007,617	850,897		156,720		
**Age (years)**					
<40	38,124	4.48	8,351	5.33	<0.0001
40–49	126,225	14.83	17,893	11.42	
50–59	221,198	26.00	34,623	22.09	
60–69	231,590	27.22	45,455	29.00	
≥70	233,760	27.47	50,398	32.16	
Male sex	425,413	50.00	72,288	46.13	<0.0001
**Diabetes duration (years)**					
<1	80,529	9.46	4,173	2.66	<0.0001
1–3	160,774	18.89	11,447	7.30	
3–5	163,043	19.16	16,812	10.73	
≥5	446,551	52.48	124,288	79.31	
Nephropathy	80,013	9.40	45,538	29.06	<0.0001
Urinary tract disease	144,668	17.00	64,407	41.10	<0.0001
Hypertension	437,205	51.38	111,952	71.43	<0.0001
Chronic obstructive pulmonary disease	103,867	12.21	40,981	26.15	<0.0001
Stroke	102,282	12.02	44,623	28.47	<0.0001
Ischemic heart disease	167,238	19.65	59,407	37.91	<0.0001
Peripheral arterial disease	81,889	9.62	40,005	25.53	<0.0001
Eye disease	46,793	5.50	36,093	23.03	<0.0001
Dyslipidemia	353,015	41.49	80,229	51.19	<0.0001
Congestive heart failure	43,318	5.09	23,155	14.77	<0.0001
Pioglitazone	18,083	2.13	7,938	5.07	<0.0001
Rosiglitazone	73,876	8.68	37,256	23.77	<0.0001
Sulfonylurea	626,050	73.58	143,176	91.36	<0.0001
Meglitinide	54,731	6.43	27,393	17.48	<0.0001
Metformin	525,367	61.74	137,423	87.69	<0.0001
Acarbose	65,988	7.76	33,249	21.22	<0.0001
Statin	176,414	20.73	50,220	32.04	<0.0001
Fibrate	156,741	18.42	44,134	28.16	<0.0001
Angiotensin-converting enzyme inhibitor/angiotensin receptor blocker	329,976	38.78	98,792	63.04	<0.0001
Calcium channel blocker	244,278	28.71	78,510	50.10	<0.0001
Aspirin	212,487	24.97	72,136	46.03	<0.0001
Non-steroidal anti-inflammatory drugs	499,863	58.75	136,735	87.25	<0.0001
Other cancer prior to baseline	78,362	9.21	18,784	11.99	<0.0001
Outpatient visits/year	12.34	25.79	17.50	20.60	<0.0001

*Outpatient visits/year is expressed as mean and standard deviation*.

Table 2 shows the incidence rates of lung cancer in never-users, ever-users, and users categorized in accordance to the tertiles of the dose-response parameters. The adjusted hazard ratio for ever-users vs. never-users was 1.545 (95% confidence interval: 1.478–1.614), suggesting a significantly higher risk of lung cancer associated with human insulin use. In the models evaluating the dose-response parameters, hazard ratios were significant in all subgroups with *P* trend < 0.0001. The hazard ratios (95% confidence intervals) were also significant for the following covariates enrolled in the model evaluating ever-users vs. never-users of insulin: age [2.460 (2.020–2.995), 4.279 (3.540–5.172), 8.355 (6.923–10.083), and 14.409 (11.941–17.387) for 40–49, 50–59, 60–69, and ≧ 70 years vs. <40 years, respectively], sex [men vs. women: 1.847 (1.789–1.907)], hypertension [0.884 (0.849–0.922)], COPD [1.413 (1.359–1.469)], eye disease [0.921 (0.869–0.976)], dyslipidemia [0.914 (0.882–0.948)], congestive heart failure [1.074 (1.011–1.140)], other cancer prior to baseline [1.593 (1.527–1.662)], metformin [0.952 (0.916–0.990)], acarbose [0.901 (0.850–0.954)], statin [0.935 (0.895–0.976)], and angiotensin-converting enzyme inhibitor and/or angiotensin receptor blocker [0.892 (0.856–0.929)].

**Table 2:** Exposure to human insulin and incidences of lung cancer and hazard ratios comparing exposed to unexposed.

**Exposure to human insulin**	**Case number**	**Incident lung cancer**	**%**	**Person-years**	**Incidence rate (per 100,000 person-years)**	**Adjusted hazard ratio**	**95% Confidence interval**	***P***
Never-users	850,897	13,677	1.61	4,361,227.25	313.60	1.000		
Ever-users	156,720	3,007	1.92	708,441.33	424.45	1.545	(1.478–1.614)	<0.0001
**TERTILE CUTOFFS**								
**Time since starting insulin (months)**								
Never-users	850,897	13,677	1.61	4,361,227.25	313.60	1.000		
<25	50,771	974	1.92	232,470.92	418.98	1.518	(1.419–1.624)	<0.0001
25–57	51,254	977	1.91	234,151.75	417.25	1.469	(1.373–1.573)	<0.0001
≥57	54,695	1,056	1.93	241,818.67	436.69	1.656	(1.549–1.772)	<0.0001
*P* trend								<0.0001
**Cumulative dosage of insulin exposure (units)**								
Never-users	850,897	13,677	1.61	4,361,227.25	313.60	1.000		
<328	51,739	1,070	2.07	245,321.33	436.16	1.368	(1.283–1.459)	<0.0001
328–16,166	51,717	979	1.89	229,593.17	426.41	1.518	(1.418–1.625)	<0.0001
≥16,166	53,264	958	1.80	233,526.83	410.23	1.902	(1.771–2.041)	<0.0001
*P* trend								<0.0001
**Cumulative duration of insulin exposure (months)**								
Never-users	850,897	13,677	1.61	4,361,227.25	313.60	1.000		
<0.57	52,245	1,105	2.12	241,256.17	458.02	1.401	(1.315–1.492)	<0.0001
0.57–8.63	51,118	944	1.85	233,903.33	403.59	1.458	(1.361–1.563)	<0.0001
≥8.63	53,357	958	1.80	233,281.83	410.66	1.945	(1.810–2.089)	<0.0001
*P* trend								<0.0001

*Hazard ratios are adjusted for all variables in Table 1*.

Table 3 shows the joint effects of insulin and COPD on the risk of lung cancer. Compared to the referent subgroup of insulin (–)/COPD (–), all the other three subgroups showed a significantly higher risk of lung cancer. Patients who had COPD and were using insulin had the highest risk, with an estimated hazard ratio of 1.891 (95% confidence interval: 1.767–2.024).

**Table 3:** Joint effects of human insulin and chronic obstructive pulmonary disease on lung cancer.

**Human insulin**	**COPD**	**Number of incident lung cancer**	**Number of cases followed**	**Adjusted hazard ratio**	**95% Confidence interval**	***P***
No	No	11,064	747,030	1.000		
Yes	No	1,937	115,739	1.383	(1.313–1.456)	<0.0001
No	Yes	2,613	103,867	1.429	(1.367–1.494)	<0.0001
Yes	Yes	1,070	40,981	1.891	(1.767–2.024)	<0.0001

*Hazard ratios are adjusted for all variables in Table 1. COPD, chronic obstructive pulmonary disease*.

Table 4 shows the subgroup analyses in patients with and without COPD. A significantly higher risk of lung cancer associated with insulin use was consistently observed, and no interaction between insulin and COPD was noted.

**Table 4:** Subgroup analyses in patients with and without chronic obstructive pulmonary disease.

**Exposure to human insulin**	**Case number**	**Incident lung cancer**	**%**	**Person-years**	**Incidence rate (per 100,000 person-years)**	**Adjusted hazard ratio**	**95% Confidence interval**	***P***
**PATIENTS WITH COPD**								
Never-users	103,867	2,613	2.52	495,698.33	527.14	1.000		
Ever-users	40,981	1,070	2.61	172,564.33	620.06	1.546	(1.430–1.673)	<0.0001
**PATIENTS WITHOUT COPD**								
Never-users	747,030	11,064	1.48	3,865,528.92	286.22	1.000		
Ever-users	115,739	1,937	1.67	535,877.00	361.46	1.550	(1.469–1.635)	<0.0001

*Hazard ratios are adjusted for all variables in Table 1. COPD, chronic obstructive pulmonary disease. P interaction between insulin and COPD = 0.2035*.

## Discussion

The findings of the present study suggested that human insulin use in patients with type 2 diabetes mellitus was associated with an increased risk of lung cancer in a dose-response pattern (Table 2). Furthermore, insulin and COPD might jointly enhance the development of lung cancer (Table 3).

The mechanisms of insulin-related lung cancer risk remain to be investigated. Some basic research provides possible explanations. An *in vitro* study using non-small-cell lung cancer cells suggested that insulin promotes the proliferation, migration, and invasion of lung cancer by increasing the phosphorylation of IR substrate 1 and activating the phosphoinositide 3-kinase/protein kinase B pathway (12). Another study showed that ablation of IR substrates 1 and 2 suppressed *Kras*-driven lung tumorigenesis (13). Because lung cancer cells may overexpress IR-A and IGF-1R, the binding of insulin to these receptors triggers the mitogenic pathways (2). Furthermore, because IGF-2 has high affinity to IR-A (2), the IGF-2 produced by cancer cells may exert an autocrine/paracrine effect by binding to IR-A and promote cancer cell proliferation (2). A study conducted in the Chinese population showed that IGF-1R was highly expressed in type 2 diabetes patients with lung cancer (14). Type 2 diabetes mellitus is characterized by insulin resistance in the metabolic pathway involving IR-B, with compensatory increase in the secretion of insulin (hyperinsulinemia). The compensatory hyperinsulinemia may overstimulate the mitogenic pathway and promote cell proliferation *via* IR-A or IGF-1R (2). Similarly, subcutaneous insulin injection for the treatment of hyperglycemia in patients with type 2 diabetes mellitus may cause hyperinsulinemia, especially in the presence of insulin resistance, which, in turn, may stimulate the mitogenic pathways involving IR-A and IGF-1R leading to new cancer development or may accelerate cell proliferation in the existence of occult lung cancer cells (2).

There are some clinical implications with these findings. First, because human insulin remains widely used for the treatment of hyperglycemia in either type 1 or type 2 diabetes patients, its potential risk of lung cancer warrants intensive investigation and immediate confirmation. Because the use of human insulin through inhalation has been shown to increase the risk of lung cancer (15), the prolonged use of insulin *via* subcutaneous injection is highly possible. Second, because this study investigated only the effect of human insulin, the findings could not be readily generalized to insulin analogs. However, because insulin glargine, the most commonly used insulin analog in Taiwan, has a higher affinity to IGF-1R (9), its potential carcinogenic effect on the lung should be highly suspected. Before human insulin, insulin glargine or other types of insulin analogs can be proven to be safe from lung carcinogenesis; the long-term use of insulin to treat diabetes should better be reserved for patients whose hyperglycemia cannot be satisfactorily treated with other anti-diabetic drugs and who are not having significant risk factors of lung cancer such as COPD as shown in the present study (Table 3). Third, the oral delivery of insulin under development for clinical use requires more intensive and cautious evaluation of cancer risk, especially of cancer involving the gastrointestinal tracts.

The present study has several strengths. First, because the NHI covers nearly the whole population in Taiwan and the longitudinal data of the patients were available throughout the period since the patients were diagnosed with diabetes until the end of 2009, the findings can be readily generalized to the whole population. Second, recall bias from self-reporting, a problem always associated with a retrospective study design, could be avoided by using the medical records. Third, detection bias in association with different socioeconomical status is less likely in this study because the NHI considers cancer as a severe morbidity and most medical co-payments can be waived. Furthermore, the drug cost-sharing in the NHI system is relatively low and can be waived in patients with low income, veterans, or those receiving prescription refills for chronic disease.

One of the major limitations of the study is the lack of information on cigarette smoking and only COPD could be used as a surrogate marker. Because the prevalence rate of COPD in ever-users of insulin was significantly higher than that in never-users of insulin (26.16 vs. 12.21%, Table 1), the subgroup analyses (Table 4) suggested that COPD might not exert a potential residual confounding effect. Furthermore, because COPD could only be partly interpreted as an indirect marker of smoking, there remained a possible confounding effect of smoking in the study if ever-users of insulin did have a significantly higher smoking rate than never-users of insulin. There was one large epidemiological study comparing the smoking rates in the Taiwanese diabetes patients during the same period (cohort enrolled with questionnaire interview from 1995 to 2002). It was noted that the prevalence rate of smoking among users of insulin was in fact lower than non-users of insulin in patients with type 2 diabetes mellitus in Taiwan. The study compared 81,923 non-users of insulin and 5,927 users of insulin and showed that the prevalence rates of smoking were 30.7% and 27.9% (*P* < 0.001), respectively (16). Therefore, if smoking played a confounding effect in the present study, this might only have underestimated the hazard ratio of lung cancer associated with insulin use.

Another limitation of the study is the lack of information on radon exposure because it is the second most important risk factor of lung cancer in the USA (17, 18). Previous studies in Taiwan suggested that radon concentrations may be high in spa water (19) and in indoor environment during cool season because of low ventilation rates (20). Therefore, inhabitants living near the source of spa water, patients who always took the spa, or patients who stayed mainly indoors during the cold season might potentially have an increased risk of lung cancer. Although studies are still lacking with regard to lung cancer risk related to radon exposure in Taiwan, the confounding effect of radon exposure should be clarified in future studies. However, because a confounder must be simultaneously associated with exposure and outcome and must not be part of the causal pathway (21), if radon exposure is not correlated with insulin use, its potential confounding in the present study may be minimal.

A third limitation is associated with the use of the reimbursement database. Many different healthcare professionals were involved in patient care, and the measurement/diagnosis of risk factors and outcome throughout the database might not be accurate and consistent compared to that achieved with a prospective cohort study. There is also a possibility that the association between risk factors and lung cancer would change with time and the presence of incomplete records might have biased the observation. This may be particularly so if the data were not missing at random (i.e., if missing data were related to the exposure of insulin use or the outcome of lung cancer). Therefore, only association and not causation can be inferred from the present study.

Other limitations included a lack of actual measurement data for confounders such as alcohol drinking, other occupational and environmental exposure, family history, exercise, lifestyle, diet, biochemical and hormonal data, genetic parameters, as well as the lack of information on the pathology, grading, and staging of lung cancer.

In conclusion, this population-based retrospective cohort study in Taiwan suggests an association between human insulin use and lung cancer. However, this should better be confirmed by additional studies in other ethnicities or by using a clinical trial. Whether insulin glargine or other insulin analogs may also increase the risk of lung cancer was not addressed here and is an issue awaiting urgent investigation.

## Data Availability

The datasets for this manuscript are not publicly available because public availability of the dataset is restricted by local regulations to protect privacy. Requests to access the datasets should be directed to C-HT, ccktsh@ms6.hinet.net.

## Ethics Statement

The study was approved by an ethic review board of the National Health Research Institutes with registered approval number 99274. The database has been de-identified for the protection of privacy and informed consent was not required according to local regulations.

## Author Contributions

C-HT researched the data and wrote the manuscript.

### Conflict of Interest Statement

The author declares that the research was conducted in the absence of any commercial or financial relationships that could be construed as a potential conflict of interest.
